# Crystal structure of bis­(diiso­propyl­ammonium) molybdate

**DOI:** 10.1107/S2056989018014755

**Published:** 2018-10-31

**Authors:** Bougar Sarr, Abdou Mbaye, Cheikh Abdoul Khadir Diop, Frederic Melin, Petra Hellwig, Mamadou Sidibé, Yoann Rousselin

**Affiliations:** a Laboratoire de Chimie Minérale et Analytique, Département de Chimie, Faculté des Sciences et Téchniques, Université Cheikh Anta Diop, Dakar, Senegal; b Laboratoire de Chimie et Physique des Matériaux (LCPM) de l’Université Assane Seck de Ziguinchor (UASZ), BP: 523 Ziguinchor, Senegal; c Chimie de la Matière Complexe UMR 7140, Laboratoire de Bioélectrochimie et Spectroscopie, CNRS-Université de Strasbourg, 1 rue Blaise Pascal, 67070 Strasbourg, France; d ICMUB-UMR 6302, 9 avenue Alain Savary, 21000 Dijon, France

**Keywords:** crystal structure, organic-inorganic salt, molybdate anion, N—H⋯O hydrogen bonding, graph set notation

## Abstract

The organic–inorganic title salt, (C_6_H_16_N)_2_[MoO_4_] or (^*i*^Pr_2_NH_2_)_2_[MoO_4_], was obtained by reacting MoO_3_ with diiso­propyl­amine in a 1:2 molar ratio in water. The molybdate anion is located on a twofold rotation axis and exhibits a slightly distorted tetra­hedral configuration. In the crystal structure, the diiso­propyl­ammmonium (^*i*^Pr_2_NH_2_)^+^ cations and [MoO_4_]^2−^ anions are linked to each other through N—H⋯O hydrogen bonds, generating rings with *R*_12_^12^(36) motifs that give rise to the formation of a three-dimensional network. The structure was refined taking into account inversion twinning (ratio of *ca* 4:1 between the two domains).

## Abstract 2

The crystal structure of the title salt, (C_6_H_16_N)_2_[MoO_4_], results from N—H⋯O hydrogen-bonded rings formed through inter­connections between the (^*i*^Pr_2_NH_2_)^+^ cations and [MoO_4_]^2−^ anions.

## Chemical context   

As a result of the photochromic properties of alkyl­ammonium molybdates (Arnaud-Neu & Schwing-Weill, 1974 ▸), molybdenum chemistry is an exciting research area. A large variety of oxidoanions based on molybdenum have been synthesized and characterized with numerous counter-cations. Among these, mononuclear and binuclear anions as well as polyoxidomolybdates with a much higher nuclearity are known (Gatehouse & Leverett, 1969 ▸; Matsumoto *et al.* 1975 ▸; Modec *et al.*, 2004 ▸; Müller & Gouzerh, 2012 ▸; Pouye *et al.*, 2014 ▸; Sarr *et al.*, 2018 ▸). Salts containing the tetra­hedral molybdate anion [MoO_4_]^2–^ combined with cations such as K^+^, Na^+^, (CH_6_N_3_)^+^, ((C_6_H_11_)_2_NH_2_)^+^, (NH_3_(CH_2_)_2_NH_3_)^+^, (OH*R*NH_3_)^+^ and (CyNH_2_)^+^ have been isolated in the past (Gatehouse & Leverett, 1969 ▸; Matsumoto *et al.*, 1975 ▸; Ozeki *et al.*, 1987 ▸; Thiele & Fuchs, 1979 ▸; Bensch *et al.*, 1987 ▸; Sheikhshoaie & Ghazizadeh, 2013 ▸; Pouye *et al.*, 2014 ▸), but never with the diiso­propyl­ammonium cation (^*i*^Pr_2_NH_2_)^+^. In a continuation of our work on molybdenum compounds with organic cations, we report here the synthesis and crystal structure of the title compound, (^*i*^Pr_2_NH_2_)_2_[MoO_4_], (I).
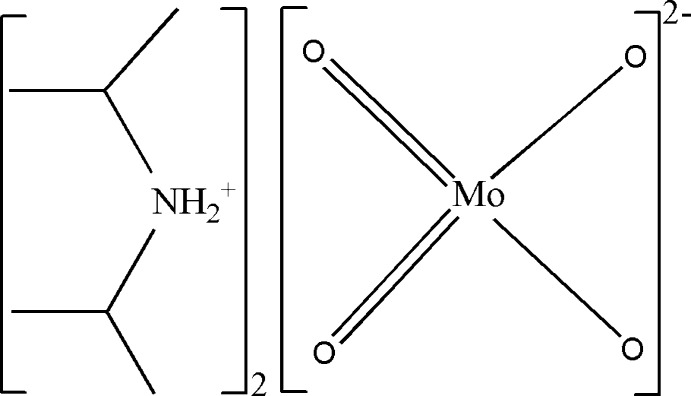


## Structural commentary   

**Figure 1:**
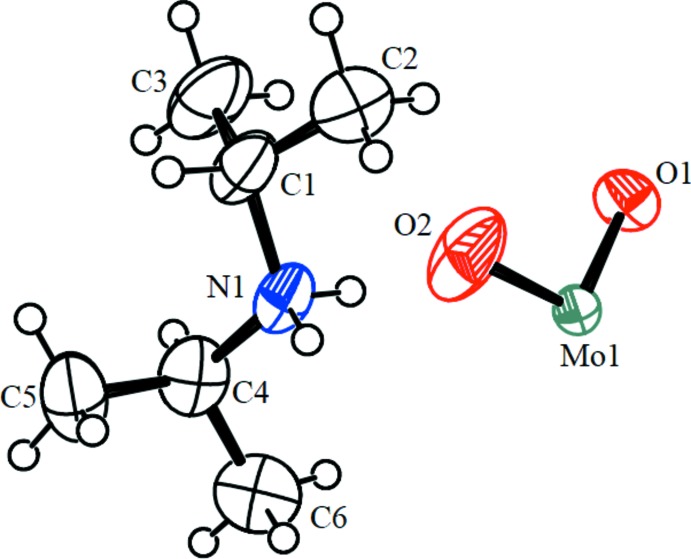
Asymmetric unit view of (I) with displacement ellipsoids drawn at the 50% probability level and hydrogen atoms as spheres of arbitrary radius.

The asymmetric unit of (I) comprises one (^*i*^Pr_2_NH_2_)^+^ cation and an {MoO_2_} entity (Fig. 1 ▸). The [MoO_4_]^2–^ molybdate anion is completed by application of twofold rotation symmetry. The two Mo—O distances are 1.732 (2) and 1.7505 (15) Å and the O—Mo—O angles vary in a narrow range between 108.77 (10) and 110.7 (2)° (Table 1 ▸), revealing only slight distortions from ideal values. Similar bond lengths and angles for the molybdate anion were reported in previous studies (Ozeki *et al.*, 1987 ▸; Bensch *et al.*, 1987 ▸; Sheikhshoaie & Ghazizadeh, 2013 ▸; Pouye *et al.*, 2014 ▸) where the Mo—O distances vary between 1.749 (2) and 1.776 (3) Å, and the O—Mo—O angles between 106.85 (4) and 113.2 (1)°.

In the crystal structure of (CyNH_2_)_2_MoO_4_·2H_2_O (Cy = cyclo­hexyl; Pouye *et al.*, 2014 ▸) the four Mo—O bond lengths are equal with 1.7613 (12) Å. Although in this structure similar N—H⋯O inter­molecular inter­actions between the (CyNH_2_)^+^ cation and the molybdate anion are present in comparison with the (^*i*^Pr_2_NH_2_)^+^ cation in the title compound, the small differences in the hydrogen-bonding pattern result in slightly different Mo—O bond lengths between the two structures. On one hand this may be related to the presence of additional water mol­ecules in (CyNH_2_)_2_MoO_4_·2H_2_O, on the other hand to steric hindrance between the four diiso­propyl­ammonium cations that surround each molybdate anion in (I). At least the strengths of the N—H⋯O hydrogen bonds do not seem to have a noticeable effect on the different Mo—O distances in (I). Both hydrogen bonds are very similar in terms of N⋯O distances and N—H⋯O angles (Table 2 ▸).

## Supra­molecular features   

**Figure 2:**
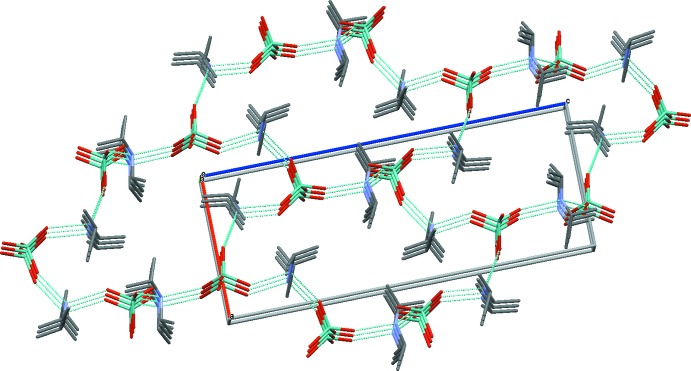
The N—H⋯O hydrogen-bonding network in (I) (turquoise dashed lines) in a view approximately along [010].

**Figure 3:**
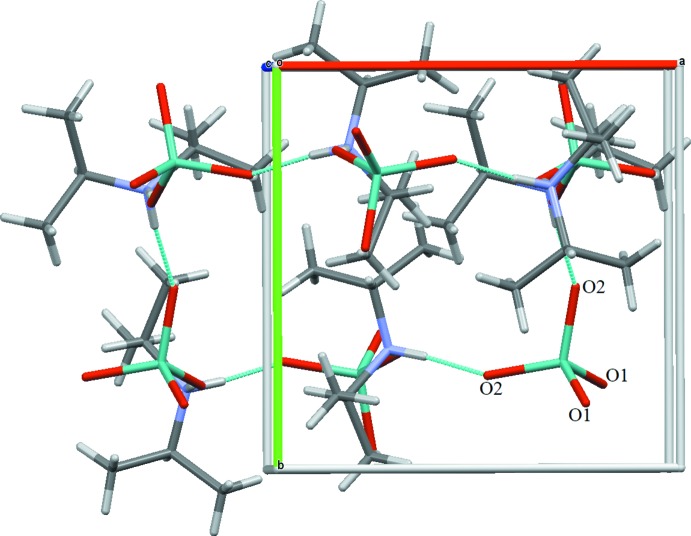
The N—H⋯O hydrogen-bonding network in (I) (turquoise dashed lines) in a view approximately along [001].

In the crystal structure of (I), each [MoO_4_]^2–^ anion is linked to two pairs of symmetry-related diiso­propyl­ammonium cations through N—H⋯O hydrogen bonds (Table 2 ▸). Contrariwise, each (^*i*^Pr_2_NH_2_)^+^ cation is linked to two molybdate [MoO_4_]^2−^ anions. The inter­action of six molybdate anions with six diiso­propyl­ammonium cations leads to {(^*i*^Pr_2_NH_2_)⋯MoO_4_}_6_ ring systems with an 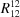
(36) motif (Etter *et al.*, 1990 ▸). Each ring is linked to six adjacent rings giving rise to infinite layers extending parallel to (010) (Fig. 2 ▸). The connection of the rings into a three-dimensional network structure perpendicular to this plane is shown in Fig. 3 ▸.

## Database survey   

A search of the Cambridge Structural Database (Version 5.39 plus 1 update, November 2017; Groom *et al.*, 2016 ▸) revealed 226 entries dealing with (^*i*^Pr_2_NH_2_)^+^ cations while 32 entries contained the [MoO_4_]^2−^ molybdate anion.

## Synthesis and crystallization   

Compound (I) was obtained from a mixture of molybdenum trioxide (3.2 g, 22.23 mmol) and diiso­propyl­amine (4 g, 44.46 mmol) in a 1:2 molar ratio in water. A clear, colourless solution was obtained after stirring for approximately one h. After twenty days of evaporation in an oven at 333 K, some colourless single crystals were obtained.

**Figure 4:**
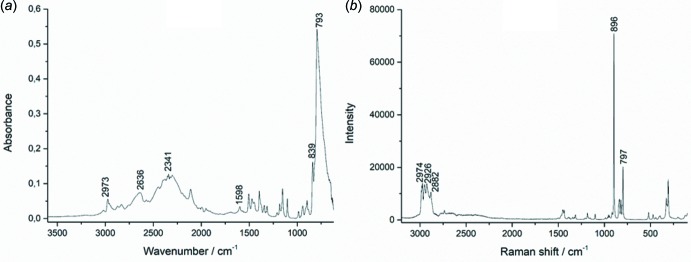
IR (*a*) and Raman (*b*) spectra of (I).

In the IR spectrum of (I) (Fig. 4 ▸ *a*), the bands at 899 and 786 cm^−1^ can be attributed to symmetric and asymmetric Mo—O stretching modes, respectively. The diso­propyl­ammonium cation is characterized by a series of vibrational bands in the 3000–2200 cm^−1^ region, which can be attributed to ν(N—H), ν(C—H) and combination modes. The δ(N—H) bending vibrations probably contribute to the signal observed at 1598 cm^−1^.

In the Raman spectrum of (I) (Fig. 4 ▸ *b*), the band at 797 cm^−1^ is attributed to the anti­symmetric stretching mode of the [MoO_4_]^2−^ molybdate anion. The symmetric vibration, ν_s_(Mo—O), in the form of a weak shoulder at 839 cm^−1^ in the infrared spectrum, is very intense in the Raman spectrum at 896 cm^−1^. In the high wavenumber region of the Raman spectrum, the bands between 3000 and 2800 cm^−1^ can be assigned to the ν(N—H) and ν(C—H) stretching vibrations of the diiso­propyl­ammonium cation.

## Refinement   

Crystal data, data collection and structure refinement details are summarized in Table 3 ▸. The structure was refined taking into account twinning by inversion (ratio of *ca* 4:1 between the two domains). H atoms were placed in geometrically idealized positions and constrained to ride on their parent atoms, with N—H distances of 0.89 Å and C—H distances of 0.96 Å for methyl and of 0.98 Å for methyl­ene groups, and with *U_iso_*(H) = 1.2*U*_eq_(C,N) or 1.5*U*_eq_(C_meth­yl_).

## Supplementary Material

Crystal structure: contains datablock(s) I. DOI: 10.1107/S2056989018014755/wm5465sup1.cif

CCDC reference: 1874215

Additional supporting information:  crystallographic information; 3D view; checkCIF report

## Figures and Tables

**Table 1:** Selected bond angles (°)

O1^i^—Mo1—O1	110.33 (12)	O2—Mo1—O1	108.77 (10)
O2—Mo1—O1^i^	109.13 (11)	O2^i^—Mo1—O2	110.7 (2)

Symmetry code: (i) 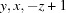.

**Table 2:** Hydrogen-bond geometry (Å, °)

*D*—H⋯*A*	*D*—H	H⋯*A*	*D*⋯*A*	*D*—H⋯*A*
N1—H1*A*⋯O2	0.89	1.80	2.684 (3)	170
N1—H1*B*⋯O1^ii^	0.89	1.81	2.695 (2)	174

Symmetry code: (ii) 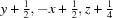.

**Table 3:** Experimental details

Crystal data	
Chemical formula	(C_6_H_16_N)_2_[MoO_4_]
*M*_r_	364.33
Crystal system, space group	Tetragonal, *P*4_3_2_1_2
Temperature (K)	293
*a*, *c* (Å)	9.0166 (1), 23.1158 (3)
*V* (Å^3^)	1879.29 (5)
*Z*	4
Radiation type	Mo *K*α
μ (mm^−1^)	0.71
Crystal size (mm)	0.38 × 0.26 × 0.1

Data collection	
Diffractometer	Rigaku Oxford Diffraction SuperNova, Dual, Cu at zero, AtlasS2
Absorption correction	Multi-scan (*CrysAlis PRO*; Rigaku OD, 2015 ▸)
*T*_min_, *T*_max_	0.612, 1.000
No. of measured, independent and observed [*I* > 2σ(*I*)] reflections	111277, 2156, 2109
*R*_int_	0.055
(sin θ/λ)_max_ (Å^−1^)	0.650

Refinement	
*R*[*F* ^2^ > 2σ(*F* ^2^)], *wR*(*F* ^2^), *S*	0.019, 0.049, 1.12
No. of reflections	2156
No. of parameters	92
H-atom treatment	H-atom parameters constrained
Δρ_max_, Δρ_min_ (e Å^−3^)	0.21, −0.31
Absolute structure	Refined as an inversion twin
Absolute structure parameter	0.19 (7)

Computer programs: *CrysAlis PRO* (Rigaku OD, 2015 ▸), *SHELXS* (Sheldrick, 2008 ▸), *SHELXL* (Sheldrick, 2015 ▸) and *OLEX2* (Dolomanov *et al.*, 2009 ▸).

## supplementary crystallographic information

### Crystal data

**Table:**

(C_6_H_16_N)_2_[MoO_4_]	*D*_x_ = 1.288 Mg m^−^^3^
*M**_r_* = 364.33	Mo *K*α radiation, λ = 0.71073 Å
Tetragonal, *P*4_3_2_1_2	Cell parameters from 49023 reflections
*a* = 9.0166 (1) Å	θ = 3.6–27.7°
*c* = 23.1158 (3) Å	µ = 0.71 mm^−^^1^
*V* = 1879.29 (5) Å^3^	*T* = 293 K
*Z* = 4	Prism, clear light colourless
*F*(000) = 768	0.38 × 0.26 × 0.1 mm

### Data collection

**Table:**

Rigaku Oxford Diffraction SuperNova, Dual, Cu at zero, AtlasS2 diffractometer	2109 reflections with *I* > 2σ(*I*)
Detector resolution: 5.3048 pixels mm^-1^	*R*_int_ = 0.055
ω scans	θ_max_ = 27.5°, θ_min_ = 3.5°
Absorption correction: multi-scan (*CrysAlis PRO*; Rigaku OD, 2015)	*h* = −11→11
*T*_min_ = 0.612, *T*_max_ = 1.000	*k* = −11→11
111277 measured reflections	*l* = −29→30
2156 independent reflections	

### Refinement

**Table:**

Refinement on *F*^2^	Hydrogen site location: inferred from neighbouring sites
Least-squares matrix: full	H-atom parameters constrained
*R*[*F*^2^ > 2σ(*F*^2^)] = 0.019	*w* = 1/[σ^2^(*F*_o_^2^) + (0.0245*P*)^2^ + 0.4149*P*] where *P* = (*F*_o_^2^ + 2*F*_c_^2^)/3
*wR*(*F*^2^) = 0.049	(Δ/σ)_max_ < 0.001
*S* = 1.12	Δρ_max_ = 0.21 e Å^−^^3^
2156 reflections	Δρ_min_ = −0.31 e Å^−^^3^
92 parameters	Absolute structure: Refined as an inversion twin
0 restraints	Absolute structure parameter: 0.19 (7)

### Special details

**Table:**

Geometry. All esds (except the esd in the dihedral angle between two l.s. planes) are estimated using the full covariance matrix. The cell esds are taken into account individually in the estimation of esds in distances, angles and torsion angles; correlations between esds in cell parameters are only used when they are defined by crystal symmetry. An approximate (isotropic) treatment of cell esds is used for estimating esds involving l.s. planes.
Refinement. Refined as a 2-component inversion twin.

### Fractional atomic coordinates and isotropic or equivalent isotropic displacement parameters (Å^2^)

**Table:**

	*x*	*y*	*z*	*U*_iso_*/*U*_eq_	
N1	0.6950 (2)	0.2985 (3)	0.57328 (7)	0.0451 (5)	
H1A	0.607999	0.280281	0.556562	0.054*	
H1B	0.681809	0.289840	0.611289	0.054*	
C1	0.8015 (3)	0.1790 (4)	0.55462 (12)	0.0603 (7)	
H1	0.897057	0.196363	0.573616	0.072*	
C2	0.7411 (5)	0.0325 (4)	0.57499 (18)	0.0827 (10)	
H2A	0.725309	0.036136	0.616035	0.124*	
H2B	0.810811	−0.044698	0.566106	0.124*	
H2C	0.648792	0.012618	0.555832	0.124*	
C3	0.8245 (4)	0.1852 (5)	0.48930 (13)	0.0872 (11)	
H3A	0.731448	0.169286	0.470093	0.131*	
H3B	0.893498	0.109574	0.477926	0.131*	
H3C	0.863030	0.280735	0.478768	0.131*	
C4	0.7349 (4)	0.4560 (3)	0.56091 (11)	0.0590 (7)	
H4	0.745520	0.468318	0.518986	0.071*	
C5	0.8813 (4)	0.4960 (4)	0.58963 (15)	0.0750 (10)	
H5A	0.876969	0.471035	0.629966	0.112*	
H5B	0.899069	0.600390	0.585479	0.112*	
H5C	0.960302	0.441624	0.571562	0.112*	
C6	0.6069 (4)	0.5518 (4)	0.58154 (14)	0.0735 (10)	
H6A	0.518209	0.524379	0.561190	0.110*	
H6B	0.628992	0.654184	0.574169	0.110*	
H6C	0.592529	0.537236	0.622295	0.110*	
Mo1	0.26396 (2)	0.26396 (2)	0.500000	0.03021 (9)	
O1	0.2086 (2)	0.1625 (2)	0.43916 (6)	0.0510 (5)	
O2	0.4506 (2)	0.2317 (4)	0.51236 (10)	0.0959 (9)	

### Atomic displacement parameters (Å^2^)

**Table:**

	*U*^11^	*U*^22^	*U*^33^	*U*^12^	*U*^13^	*U*^23^
N1	0.0337 (9)	0.0747 (15)	0.0269 (8)	0.0005 (9)	−0.0009 (7)	−0.0005 (9)
C1	0.0350 (13)	0.095 (2)	0.0510 (15)	0.0047 (13)	0.0011 (11)	−0.0167 (15)
C2	0.069 (2)	0.075 (2)	0.104 (3)	0.006 (2)	0.010 (2)	−0.016 (2)
C3	0.070 (2)	0.138 (3)	0.0536 (17)	0.007 (2)	0.0170 (16)	−0.026 (2)
C4	0.0638 (19)	0.0767 (18)	0.0364 (12)	−0.0047 (17)	−0.0013 (14)	0.0133 (12)
C5	0.064 (2)	0.083 (3)	0.078 (2)	−0.0223 (18)	−0.0017 (18)	0.003 (2)
C6	0.086 (2)	0.071 (2)	0.0631 (18)	0.0121 (18)	−0.0156 (17)	0.0083 (17)
Mo1	0.03380 (10)	0.03380 (10)	0.02304 (12)	−0.00356 (9)	−0.00072 (6)	0.00072 (6)
O1	0.0687 (12)	0.0566 (10)	0.0277 (7)	−0.0072 (9)	−0.0063 (7)	−0.0049 (7)
O2	0.0350 (9)	0.178 (3)	0.0751 (14)	0.0026 (14)	−0.0119 (9)	−0.0308 (18)

### Geometric parameters (Å, º)

**Table:**

N1—H1A	0.8900	C4—H4	0.9800
N1—H1B	0.8900	C4—C5	1.521 (5)
N1—C1	1.506 (4)	C4—C6	1.518 (5)
N1—C4	1.493 (4)	C5—H5A	0.9600
C1—H1	0.9800	C5—H5B	0.9600
C1—C2	1.504 (5)	C5—H5C	0.9600
C1—C3	1.525 (4)	C6—H6A	0.9600
C2—H2A	0.9600	C6—H6B	0.9600
C2—H2B	0.9600	C6—H6C	0.9600
C2—H2C	0.9600	Mo1—O1	1.7505 (15)
C3—H3A	0.9600	Mo1—O1^i^	1.7504 (15)
C3—H3B	0.9600	Mo1—O2	1.732 (2)
C3—H3C	0.9600	Mo1—O2^i^	1.732 (2)
			
H1A—N1—H1B	107.1	N1—C4—H4	108.7
C1—N1—H1A	107.8	N1—C4—C5	110.5 (2)
C1—N1—H1B	107.8	N1—C4—C6	107.3 (3)
C4—N1—H1A	107.8	C5—C4—H4	108.7
C4—N1—H1B	107.8	C6—C4—H4	108.7
C4—N1—C1	118.2 (2)	C6—C4—C5	112.9 (3)
N1—C1—H1	108.5	C4—C5—H5A	109.5
N1—C1—C3	110.1 (3)	C4—C5—H5B	109.5
C2—C1—N1	108.0 (3)	C4—C5—H5C	109.5
C2—C1—H1	108.5	H5A—C5—H5B	109.5
C2—C1—C3	113.0 (3)	H5A—C5—H5C	109.5
C3—C1—H1	108.5	H5B—C5—H5C	109.5
C1—C2—H2A	109.5	C4—C6—H6A	109.5
C1—C2—H2B	109.5	C4—C6—H6B	109.5
C1—C2—H2C	109.5	C4—C6—H6C	109.5
H2A—C2—H2B	109.5	H6A—C6—H6B	109.5
H2A—C2—H2C	109.5	H6A—C6—H6C	109.5
H2B—C2—H2C	109.5	H6B—C6—H6C	109.5
C1—C3—H3A	109.5	O1^i^—Mo1—O1	110.33 (12)
C1—C3—H3B	109.5	O2^i^—Mo1—O1	109.13 (11)
C1—C3—H3C	109.5	O2—Mo1—O1^i^	109.13 (11)
H3A—C3—H3B	109.5	O2—Mo1—O1	108.77 (10)
H3A—C3—H3C	109.5	O2^i^—Mo1—O1^i^	108.77 (10)
H3B—C3—H3C	109.5	O2^i^—Mo1—O2	110.7 (2)
			
C1—N1—C4—C5	−59.1 (3)	C4—N1—C1—C2	176.6 (2)
C1—N1—C4—C6	177.5 (2)	C4—N1—C1—C3	−59.5 (3)

Symmetry code: (i) *y*, *x*, −*z*+1.

### Hydrogen-bond geometry (Å, º)

**Table:**

*D*—H···*A*	*D*—H	H···*A*	*D*···*A*	*D*—H···*A*
N1—H1*A*···O2	0.89	1.80	2.684 (3)	170
N1—H1*B*···O1^ii^	0.89	1.81	2.695 (2)	174

Symmetry code: (ii) *y*+1/2, −*x*+1/2, *z*+1/4.
